# High Performance Phototransistor Based on 0D-CsPbBr_3_/2D-MoS_2_ Heterostructure with Gate Tunable Photo-Response

**DOI:** 10.3390/nano15040307

**Published:** 2025-02-17

**Authors:** Chen Yang, Yangyang Xie, Lei Zheng, Hanqiang Liu, Peng Liu, Fang Wang, Junqing Wei, Kailiang Zhang

**Affiliations:** Tianjin Key Laboratory of Film Electronic & Communication Devices, School of Integrated Circuit Science and Engineering, Tianjin University of Technology, Tianjin 300384, China; yangc0711@163.com (C.Y.); leizheng@tju.edu.cn (L.Z.); hanqiang_liu@163.com (H.L.); angelheart0802@163.com (P.L.); fwang75@email.tjut.edu.cn (F.W.)

**Keywords:** MoS_2_, CsPbBr_3_ perovskite quantum dots, phototransistor, gate tunable photo-response

## Abstract

Monolayer MoS_2_ has been widely researched in high performance phototransistors for its high carrier mobility and strong photoelectric conversion ability. However, some defects in MoS_2_, such as vacancies or impurities, provide more possibilities for carrier recombination; thus, restricting the formation of photocurrents and resulting in decreased responsiveness. Herein, all-inorganic CsPbBr_3_ perovskite quantum dots (QDs) with high photoelectric conversion efficiency and light absorption coefficients are introduced to enhance the responsivity of a 2D MoS_2_ phototransistor. The CsPbBr_3_/MoS_2_ heterostructure has a type II energy band, and it has a high responsivity of ~1790 A/W and enhanced detectivity of ~2.4 × 10^11^ Jones. Additionally, the heterostructure CsPbBr_3_/MoS_2_ enables the synergistic effect mechanism of photoconduction and photogating effects with the gate tunable photo-response, which could also contribute to an improved performance of the MoS_2_ phototransistor. This work provides new strategies for performance phototransistors and is expected to play an important role in many fields, such as optical communication, environmental monitoring and biomedical imaging, and promote the development and application of related technologies.

## 1. Introduction

A heterostructure built using two-dimensional materials with strong light absorption ability can be used when designing high performance photoelectronics, such as phototransistors and photodiodes [1,2,3]. Two of the most popular approaches are building photodiodes using two different 2D materials and phototransistors based on 2D semiconductors as channels [4,5].

Transition metal dichalcogenides (TMDCs)-MoS_2_ have shown great potential in fabricating photodetectors, including phototransistors and photodiodes, in the last decade [6,7,8,9,10]. M. Park et al. constructed vertical WSe_2_/MoS_2_ heterojunctions to enhance the responsivity of near-infrared light. The responsivity reached 0.3 A/W thanks to the highly effective generation, separation and transportation of photocarriers [11]. In 2021, Y. Mao et al. built lateral MoS_2_ Schottky photodetectors and the maximum responsivity reached 1.9 A/W (wavelength~1310 nm). A gold electrode absorbs most of the incident light through the transparent MoS_2_ layers, and a photocurrent gain is generated in the MoS_2_ layers [12]. In 2024, L. Wang et al. used tellurium because the (Te) microwire and polyvinyl alcohol (PVA) combine to form a functional flexible substrate to improve the detection performance of an MoS_2_ device in near-infrared range. A responsivity of 0.28 mA/W and a detectivity of similar to 1.41 × 10^10^ Jones were realized by using a 808 nm laser [13].

However, most MoS_2_ photoelectronic devices are mainly targeted at visible and near-infrared light absorption or detection caused by narrow bandgap of MoS_2_ (1.2–1.8 eV), which limit their further application in photoelectronics [14]. It has been found that 0D quantum dots (QDs) showed promising absorption ability in a wide range spectrum and they could be easily integrated with two-dimensional materials to form a van der waals heterostructure for designing high performance optoelectronics [15]. Konstantatos et al. used PbS quantum dots as a photosensitive layer to build a PbS/graphene photodetector, which achieved an increased responsivity from 10^−2^ A/W to 10^7^ A/W [16]. Next, the researchers set out to combine different quantum dots with TMDCs to demonstrate the role of quantum dots in improving photoelectric performance. They could modulate the charge density in TMDC-based optoelectronic devices when illuminated with light [17,18,19]. In 2018, Sangyeon Pak et al. prepared an MoS_2_/PbS QDs heterostructure phototransistor and the detectivity reached 1 × 10^11^ Jones because of the enhanced generation of photoexcited carriers in PbS QDs [20]. In addition, all-inorganic halogen perovskites with the molecular formula CsPbX_3_ (X = Cl, Br, I) showed more competitive prospects in new optoelectronic application due to their adjustable band gap, expected absorption efficiency and easy fabrication process [21,22,23,24,25]. And it was a promising strategy to integrate CsPbX_3_ with a high carrier transport layer and a high light absorption layer for constructing optoelectronic devices with improved photon–electron conversion efficiency.

In this work, we designed and fabricated a heterostructure phototransistor by using MoS_2_ nanosheet and CsPbBr_3_ QDs. Here, monolayer MoS_2_ nanosheets synthesized by chemical vapor deposition (CVD) method were used as the conductive channel of the phototransistor. CsPbBr_3_ QDs were spin-coated on MoS_2_ nanosheets as the light absorption layer. The device exhibited an enhanced responsivity (1790 A/W) and a higher detectivity (2.4 × 10^11^ Jones) in ultraviolet light compared to a bare MoS_2_ phototransistor. Here, the generated photoelectron–hole pairs in CsPbBr_3_ would separate and the electrons would rapidly inject into MoS_2_, which reduced the charge recombination and accelerated the transport of the carriers [26,27]. The increased photocurrent and improved photo-response resulted from the synergistic effect of both photoconduction and photogating in the CsPbBr_3_/MoS_2_ heterostructure phototransistor. This work laid the foundation for the development of high performance optoelectronics based on 2D materials and 0D perovskite.

## 2. Materials and Methods

### 2.1. Fabrication of CsPbBr_3_ QDs

**Figure 1:**
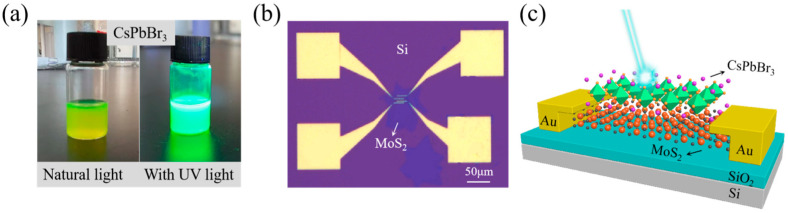
Materials and device preparation: (**a**) CsPbBr_3_ QDs solution with normal light and UV light excitation; (**b**) optical image of MoS_2_-transistor.; and (**c**) diagram of the CsPbBr_3_/MoS_2_ heterostructure transistor.

For CsPbBr_3_ fabrication, CsBr (0.1 mmol, Beijing Yinokai Technology, China), PbBr_2_ (0.1 mmol, Shanghai McLean Biochemical Technology, China) and 10 mL DMF (Beijing Yinokai Technology, China) solution were first mixed at room temperature, then oleic acid (0.25 mL, Tianjin Xinsi Biochemical Technology, China) and oleamine (0.13 mL, Tianjin Xinsi Biochemical Technology, China) were added to stabilize the precursor solution. Stirring continued until all the precursor powders were completely dissolved into a suspension. Finally, 1 mL precursor suspension and 10 mL xylene (Tianjin Kemi Ou chemical reagent, China) were mixed and we obtained CsPbBr_3_ QDs solution. It could be seen that with natural light excitation, the quantum dot solution emitted yellow light and when using ultraviolet light as excitation source, the quantum dot solution emitted green light (Figure 1a).

### 2.2. Fabrication of Monolayer MoS_2_ Nanosheets

MoS_2_ nanosheets were fabricated on a SiO_2_/Si substrate (Suzhou silicon electronic Technology, China) using the NaCl assisted CVD method, with MoO_3_ (Alfa Aesar, China) and S (Alfa Aesar, China) powder as precursors and NaCl as the catalyst. Here, the mass ratio of MoO_3_/NaCl was 1/2 (wt%), and Ar was used as carrier gas with a flow of 20 sccm. The deposition process was achieved in a double temperature zone tube furnace, the reaction temperature was set to ~750 °C and remained for 30–35 min, ensuring the full reaction of all precursors.

### 2.3. Fabrication and Measurements of CsPbBr_3_/MoS_2_ Phototransistor

First, the MoS_2_ nanosheets were transferred onto a new SiO_2_/Si substrate (0.05 Ω·cm) and used as conductive channel; e-beam lithography was implemented, followed by evaporating Ti/Au (10/80 nm) as the source/drain electrode. After that, CsPbBr_3_ QDs solution was fabricated on MoS_2_ to form a CsPbBr_3_/MoS_2_ heterostructure (Figure 1b). All electrical properties were measured using a semiconductor parameter analyzer (Agilent, B1500, Santa Clara, CA, USA) and a 405 nm wavelength laser (Figure 1c).

## 3. Results

### 3.1. Characterizations of CsPbBr_3_/ MoS_2_ Heterostructure

**Figure 2:**
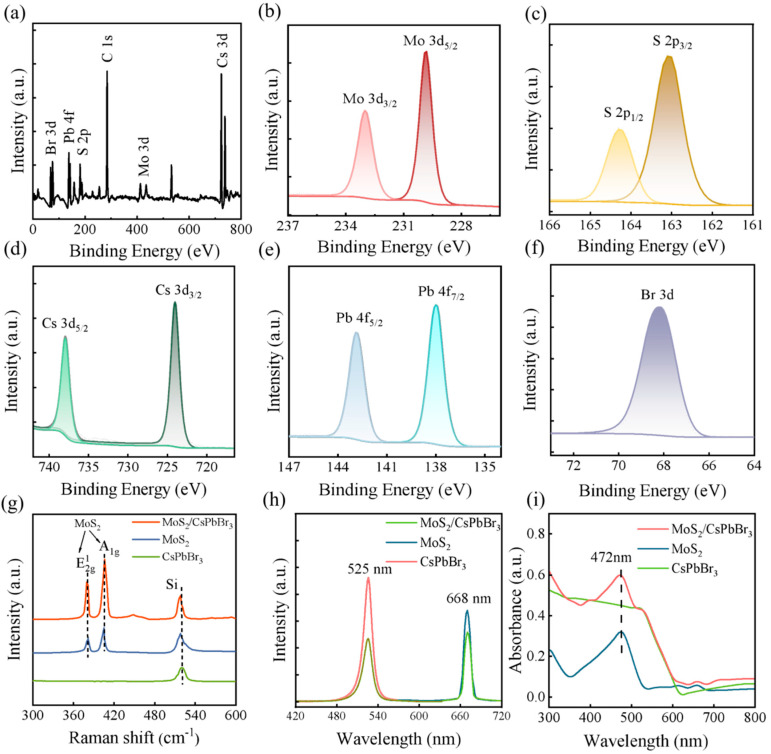
Characterizations of bare MoS_2_ nanosheets, CsPbBr_3_ QDs and CsPbBr_3_/MoS_2_ heterostructure: (**a**) XPS full-spectrum; (**b**–**f**) specific XPS spectrums of Mo, S, Cs, Pb and Br elements; (**g**) Raman spectrum; (**h**) PL spectrum; and (**i**) absorption spectrum.

The chemical stability of each layer in the CsPbBr_3_/MoS_2_ heterostructure was first proved by XPS test. Typical peaks of Mo 3d, S 2p, Cs 3d, Pb 4f and Br 3d were all observed in the wide-scan spectrum, as shown in Figure 2a. The characteristic peaks of Mo 3d were located at 233 and 223 eV (Figure 2b), S 2p at 164 and 163 eV (Figure 2c), Cs 3d at 734 and 724 eV (Figure 2d), Pb 4f at 143 and 138 eV (Figure 2e) and Br 3d at 68.5 eV (Figure 2f), respectively. The results were highly consistent with bare MoS_2_ and CsPbBr_3_ QDs, suggesting the high stability of both CsPbBr_3_ and MoS_2_ in the heterostructure. Meanwhile, the molecular structural stability of MoS_2_, CsPbBr_3_ QDs and the CsPbBr_3_/MoS_2_ heterostructure was evaluated by Raman characterization with an excitation laser of 532 nm (5 mW). Here, ${E\;}_{2g}^{1}$ and $A_{1g}$ characteristic peaks of MoS_2_ were located at 380.54 cm^−1^ and 403.73 cm^−1^, respectively, while the original CsPbBr_3_ QDs showed no obvious Raman peaks. In the CsPbBr_3_/MoS_2_ heterostructure, the Raman peaks were still dominated (Figure 2g) by MoS_2_ and there were no obvious shifts compared to bare MoS_2_, indicating that MoS_2_ had high quality without any damage during the preparation process.

The fabricated MoS_2_ had a clean surface and specific triangle topography with thickness of ~0.74 nm (monolayer, Figure S1). The PL spectra of bare monolayer MoS_2_ (668 nm), bare CsPbBr_3_ QDs (525 nm) and the CsPbBr_3_/MoS_2_ heterostructure were excited by a 405 nm laser (Figure 2h). As expected, the PL peak of the CsPbBr_3_/MoS_2_ heterostructure showed excitation peaks both in 525 (CsPbBr_3_ QDs) and 668 nm (MoS_2_), suggesting independent photo-generated carriers and photon emission in each layers. There was a large drop in PL intensity of CsPbBr_3_/MoS_2_ compared to bare MoS_2_ nanosheets or CsPbBr_3_ QDs, which might be caused by the effective carrier transport from CsPbBr_3_ to MoS_2_; it also proved that CsPbBr_3_ as a photosensitive layer could indeed realize the effective separation of carriers. On the other hand, the PL peaks of all layers exhibited a narrow FWHM, suggesting a high crystal quality.

Furthermore, the light absorption capability of MoS_2_, CsPbBr_3_ QDs and the CsPbBr_3_/MoS_2_ heterostructure were tested and the corresponding ultraviolet-visible (UV-vis) spectra are shown in Figure 2i. The absorption intensity of the CsPbBr_3_/MoS_2_ heterostructure was significantly increased compared with bare MoS_2_ or CsPbBr_3_. In particular, the CsPbBr_3_/MoS_2_ heterostructure contained the absorption peaks of both MoS_2_ and CsPbBr_3_, and significantly enhanced at the range of 400~500 nm and 617~661 nm, which might be due to the formation of new electronic states and band structures in the CsPbBr_3_/MoS_2_ heterostructure. At the same time, the similar absorption capacity of the CsPbBr_3_/MoS_2_ heterostructure and bare CsPbBr_3_ QDs confirmed the effective photosensitive layer role of CsPbBr_3_ in this heterostructure.

**Figure 3:**
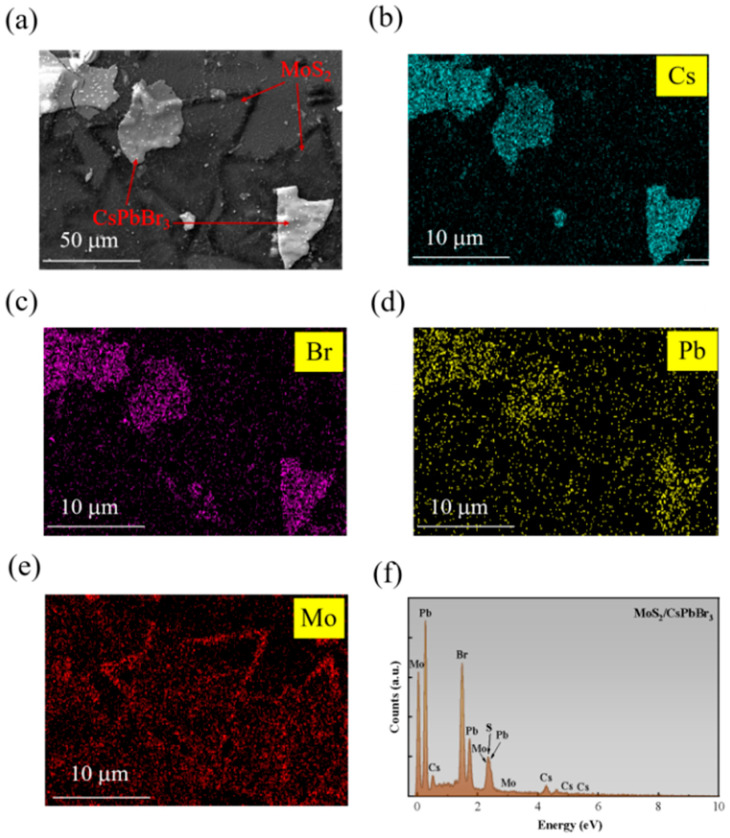
Component element analysis of CsPbBr_3_/MoS_2_: (**a**) SEM image; (**b**–**e**) the mapping images of Mo, Cs, Pb and Br elements, respectively; and (**f**) intensities of all the elements according to EDS.

From Figure 3a, the CsPbBr_3_ nanocrystals uniformly distributed on the MoS_2_ nanosheet and Cs, Pb, Br and Mo elements could be observed from the energy dispersive spectrum (EDS) mapping in Figure 3b–e, respectively. The specific element ratio according to Figure 3f further verified the contained elements of this CsPbBr_3_/MoS_2_ var der waals heterostructure.

### 3.2. Electrical Performance of CsPbBr_3_/MoS_2_ Phototransistor

**Figure 4:**
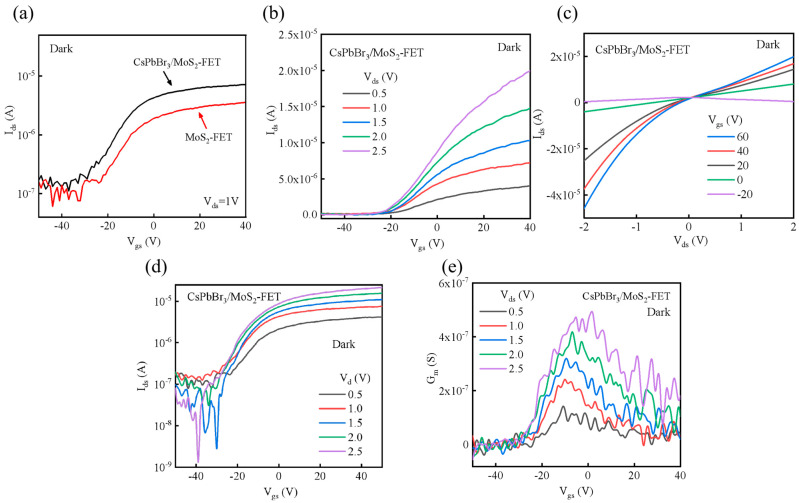
Electrical properties of CsPbBr_3_/MoS_2_ phototransistor without light (dark): (**a**) performance comparison of bare MoS_2_ and CsPbBr_3_/MoS_2_ phototransistor; (**b**) transfer curves; (**c**) output curves; (**d**) transfer curves in logarithmic scale; and (**e**) transconductance curves.

The electrical performance of CsPbBr_3_/MoS_2_ and the bare MoS_2_ phototransistor under dark conditions is shown in Figure 4a. The current of the CsPbBr_3_/MoS_2_ heterostructure phototransistor was increased by nearly 25% compared to bare MoS_2_ transistor. This might be because of the spontaneous transfer of electrons from MoS_2_ to the CsPbBr_3_ layer. Figure 4b shows the transfer characteristic curves of the CsPbBr_3_/MoS_2_-phototransistor (*I_ds_* − *V_gs_*), which was similar to the bare MoS_2_-FET. The almost linear relationship of the out-put characteristic curves in Figure 4c indicated good ohmic contact between the metal electrode and the conducting channel. The device showed an acceptable on/off ratio of ~10^3^ according to Figure 4d. The carrier mobility was calculated by Formula (1),

(1) $$\mu =\frac{{dI}_{ds}}{{dV}_{gs}}\times \frac{L}{W}\times \frac{1}{C_{g}V_{gs}}$$

where *W*/*L* is the channel width/length, and *C_g_* is the capacitance of the dielectric layer [28]. Here, *L*/*W* are 2/5, respectively, and the calculated mobility is 2.32 cm^2^ V^−1^s^−1^. The transconductance in Figure 4e increased first and reached the maximum value, as the carrier concentration increased according to the gate voltage. Then it gradually reduced with the increasing gate voltage because of the saturation of the carriers.

### 3.3. Photoelectrical Performance of CsPbBr_3_/MoS_2_ Phototransistor

**Figure 5:**
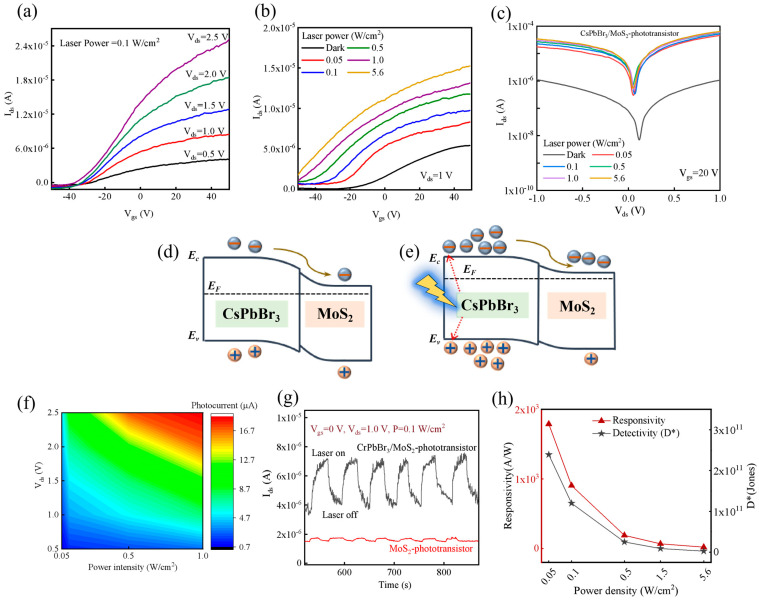
Response of CsPbBr_3_/MoS_2_ phototransistor with illumination: (**a**) photocurrent of the device with laser power of 0.1 W/cm^2^; (**b**) photocurrent of the device with different laser power; (**c**) output characteristic curve with different laser power at *V_gs_* = 20 V; (**d**,**e**) schematic diagram of charge generation and transport of CsPbBr_3_/MoS_2_ phototransistor under dark and laser irradiation, respectively; (**f**) photocurrent mapping with different laser power intensity and V_ds_; (**g**) optical switching characteristics; and (**h**) responsivity and detectivity of CsPbBr_3_/MoS_2_ phototransistor.

Further, we revealed the photosensitivity of the CsPbBr_3_/MoS_2_ phototransistor by using a 405 nm laser as the illumination source. The photocurrent (laser power = 0.1 W/cm^2^) of the device steadily increased, with V_ds_ increasing from 0.5 to 2.5 V, indicating that the photo-generated carriers mainly contributed to an increase in the channel current (*I_ds_*) in this device under illumination (Figure 5a). Then we studied the device performance using different laser power (0.05~5.6 W/cm^2^, V_ds_ = 1 V) in Figure 5b. The threshold voltage of the CsPbBr_3_/MoS_2_ phototransistor gradually moved towards the negative direction as the laser power increased. This might be attributed to the trapping of photo-generated holes at the interface of CsPbBr_3_/MoS_2_, which electrostatically modulated the carrier. When the laser power differs, the photocurrent is affected (Figure 5c). The channel current illuminated by the external light was higher than that under dark conditions, and it further increased according to the incident light power, which implied that the photocurrent was dominated by the photoconduction effect, in the CsPbBr_3_/MoS_2_ heterostructure phototransistor in this case.

From the band alignment diagram of the CsPbBr_3_/MoS_2_ phototransistor under dark and light conditions, it could be seen that under dark conditions (Figure 5d), a depletion layer was formed due to the difference in the Fermi levels of MoS_2_ and CsPbBr_3_, and only a small number of electrons transferred spontaneously from CsPbBr_3_ to MoS_2_; thus, the current was weak. After illumination, a large number of photo-generated carriers (electron–hole pairs) would be produced in CsPbBr_3_ (Figure 5e), and the internal electric field would drive more electrons transfer from CsPbBr_3_ to MoS_2_, greatly increasing the photocurrent [29].

The relationship between drain voltage, power density and photocurrent were obtained (Figure 5f). The fabricated heterostructure phototransistor had higher *I_ds_* at V_ds_ and increasing laser power, suggesting the CsPbBr_3_/MoS_2_ heterostructure had good light absorption capability and thus, contributed to a higher photo-response. A transient light-response in Figure 5g shows an obvious switching characteristic of the CsPbBr_3_/MoS_2_ phototransistor under a fixed laser power of 0.1 W/cm^2^ and stable photocurrent with low dark current.

The photocurrent was calculated by Formula (2),

(2) $$I_{ph}=I_{light}-I_{dark}$$

where *I_dark_* is 1.5 × 10^−5^ A (*V_gs_* = 20 V). Typically, we evaluated light absorption (R) of the phototransistor by Formula (3),

(3) $$R=\frac{I_{ph}}{P\times S}$$

where P is the incident light power intensity, S is the effective irradiation area of the device and the effective area S is 4 μm^2^ in this work. Responsivity (R) of the CsPbBr_3_/MoS_2_ phototransistor decreased as the laser power increased from 0.05 to 5.6 W (Figure 5h). Specific detectivity (D*) was calculated according to Formula (4),

(4) $$D*=\frac{R\sqrt{S}}{\sqrt{2eI_{dark}}}$$

where e is the electron charge and S is the effective irradiation area of the device. The results showed that R and D* reached the maximum value of 1790 A/W and 2.4 × 10^11^ Jones, respectively, with a 405 nm laser (intensity of 0.05 W/cm^2^), which were significantly improved compared to bare MoS_2_ phototransistor (330 A/W and 4.4 × 10^10^ Jones in Figure S2).

**Figure 6:**
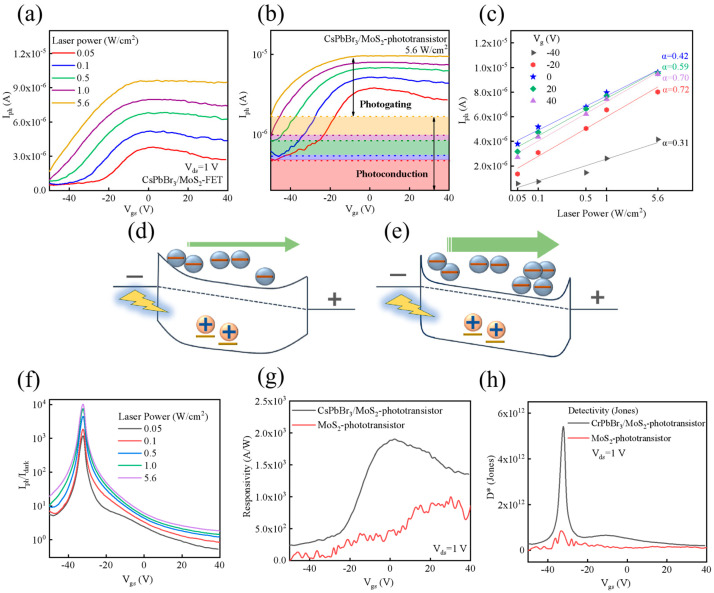
The device performance of CsPbBr_3_/MoS_2_ phototransistor: (**a**) photocurrent under laser irradiation with different light power intensities; (**b**) gate voltage dependence of photocurrent; (**c**) photocurrent conformed to a simple power law of laser intensity; (**d**,**e**) band diagram of CsPbBr_3_/MoS_2_ phototransistor for photoconduction and photogating; (**f**) gate voltage dependence of *I_ph_*/*I_dark_*; (**g**) responsivity of CsPbBr_3_/MoS_2_ and bare MoS_2_ phototransistor; and (**h**) detectivity of both two types of the devices.

The change in photocurrent under different gate voltage (Figure 6a) showed that the photocurrent was closely related to the gate voltage and optical power. Under illumination, the photo-generated electron–hole pairs in CsPbBr_3_ were separated and then transferred to MoS_2_, forming the photocurrent (*I_ph_*). When the light intensity increased, the *I_ph_* of the CsPbBr_3_/MoS_2_ phototransistor also increased correspondingly, as the photo-generated carriers increased with the increasing laser power, indicating a photoconduction effect. Moreover, the *I_ph_* of the CsPbBr_3_/MoS_2_ phototransistor increased when the gate voltage swept from −50 to −10 V (Figure 6b), and then saturated at ~−10 V. Power law fitting was carried out through *I_ph_*~P^α^ (Figure 6c) and α was the slope. The photocurrent had a linear relationship with the incident light power (i.e., α = 1), indicating the photocurrent was proportional to the incident light power and the photoconduction effect was dominated in the device [30]. When 0 < α < 1, the photocurrent had a nonlinear relation with the incident light power. At this time, with the gate voltage decreasing from −40 to 40 V, the dependence of the photocurrent on the incident power increased nonlinearly, indicating that the photogating effect dominated the generated photocurrent at this time.

The gate-modulated effects in Figure 6d describe the electron–hole pair generated when CsPbBr_3_ absorbs photons during illumination. Due to the difference in Fermi levels between CsPbBr_3_ and MoS_2_, the depletion layer provided a built-in electric field that promoted the transfer of electrons from CsPbBr_3_ to the MoS_2_ layer and thus, increased the carrier concentration and *I_ph_* (photoconduction). Meanwhile, those holes were limited in the CsPbBr_3_ layer and generated a positive gate effect towards the MoS_2_ channel. As the gate voltage increased (Figure 6e), more carriers were generated in the MoS_2_ channel; thus, contributing to an enhanced photogating effect, as well as increased *I_ph_*, which would saturate as the gate voltage continually increased.

Alternatively, while *I_ph_*/*I_dark_* was obviously regulated by the gate voltage (Figure 6f), it also identified the influence of the photogating effect on *I_ph_*. Both responsivity (R, Figure 6g) and detectivity (D*, Figure 6h) of the CsPbBr_3_/MoS_2_ phototransistor were improved compared to the bare MoS_2_ device, and they were strongly associated with the gate voltage, e.g., R reached its maximum value at ~0 V (V_g_) and D* showed a peak value at ~−30 V (V_g_). Additionally, the special band gap of MoS_2_ and CsPbBr_3_ might have promoted the transfer of electrons between the van der waals layers and, we realized, a phototransistor with violet light absorption capability, high responsivity and detectivity (Table 1).

Table 1 summarizes the detection bands, responsivity and detectivity of phototransistors with different structures based on the MoS_2_ materials reported in recent years. It can be found that the 0D perovskite quantum dots/MoS_2_ heterostructure phototransistor has effectively improved the responsivity in this work. And the photocurrent is increased by nearly 20% with ultraviolet light as an excitation source, which provides certain data support for the research of two-dimensional material optoelectronic devices.

## 4. Discussion

In summary, we demonstrated a high-performance phototransistor by integrating a high carrier transport layer (2D-MoS_2_) and an outstanding photo absorption layer (CsPbBr_3_ QDs). The fabricated CsPbBr_3_/MoS_2_ phototransistor had a wide absorption spectrum between 300 and 800 nm. In addition, the decline in PL peaks also demonstrated the effective transfer of charge from CsPbBr_3_ to MoS_2_. Due to the high absorptivity and effective interfacial charge separation of the CsPbBr_3_/MoS_2_ heterostructure phototransistor, we obtained an increased responsivity of 1790 A/W and a detectivity of 2.4 × 10^11^ Jones. The mechanisms of photocurrent generation and regulation were also identified by modulating the laser power and gate voltage. Both photoconduction and photogating effects were present and synergistically contributed to the generation of photocurrent, and photogating effects became dominant with the higher gate voltage. The results showed that the coupling of 2D nanomaterials with the perovskite layer was an ideal choice to realize the next generation of high-performance phototransistor.

## Figures and Tables

**Table 1:** The responsivity and detectivity of various phototransistors based on 2D materials.

Structure	MoS_2_ Layer	Wavelength (nm)	Responsivity (A/W)	Detectivity (Jones)	Ref.
MoS_2_	monolayer	640	50	-	[31]
MoS_2_	multilayer	633	0.12	10^10^	[32]
MoS_2_/MoTe_2_	few layers	637	4.6 × 10^−2^	1.06 × 10^7^	[33]
MoS_2_/CH_3_NH_3_PbI_3_	few layers	638	1.1	9 × 10^10^	[34]
MoS_2_/2DPI	monolayer	900	390.5	5.10 × 10^12^	[35]
CsPbBr_3_/MoS_2_	monolayer	405	1790	2.39 × 10^11^	This study

## Data Availability

Data is contained within the article or Supplementary Material.

## Supplementary Materials

The following supporting information can be downloaded at: https://www.mdpi.com/article/10.3390/nano15040307/s1, Figure S1. Topography of monolayer MoS2. (a) light image. (b) AFM and step test. Figure S2. Device performance of bare MoS2 phototransistor irradiating by a 405 nm laser with different power density. (a) Transfer characteristics with V_ds_ = 1 V. (b) Output characteristics with *V_gs_* = 20 V. (c) The intrinsic photocurrent under light irradiation. (d) Photo-switching characteristics. (e) The calculated responsivity and detectivity.
