# Supplementary material for: High Performance Phototransistor Based on 0D-CsPbBr3/2D-MoS2 Heterostructure with Gate Tunable Photo-Response
Source: Nanomaterials (Basel). 2025 Feb 17;15(4):307. doi: 10.3390/nano15040307 (PMC11858019; doi:10.3390/nano15040307)
Supplement: Supplementary file 1 [file nanomaterials-15-00307-s001.zip › nanomaterials-3472871-supplementary.pdf]

Supplementary data

High Performance Phototransistor Based on 0D-$CsPbBr_3$/2D-MoS_2 Heterostructure with Gate Tunable Photo-Response

Chen Yang, Yangyang Xie *, Lei Zheng, Hanqiang Liu, Peng Liu, Fang Wang, Junqing Wei * and Kailiang Zhang *

Tianjin Key Laboratory of Film Electronic & Communication Devices, School of Integrated Circuit Science and Engineering, Tianjin University of Technology, Tianjin 300384, China; yangc0711@163.com (C.Y.); leizheng@tju.edu.cn (L.Z.); hanqiang_liu@163.com (H.L.); angelheart0802@163.com (P.L.); fwang75@email.tjut.edu.cn (F.W.)

* Correspondence: xyyhebut@163.com (Y.X.); weijunging0907@163.com (J.W.); kailiang_zhang2007@163.com (K.Z.); Tel.: +86-136-7219-8378 (K.Z.)

As-fabricated MoS_2 nanosheet was shown in Figure S1, it had clean surface and good topography with a thickness of ~ 0.74 nm (monolayer).

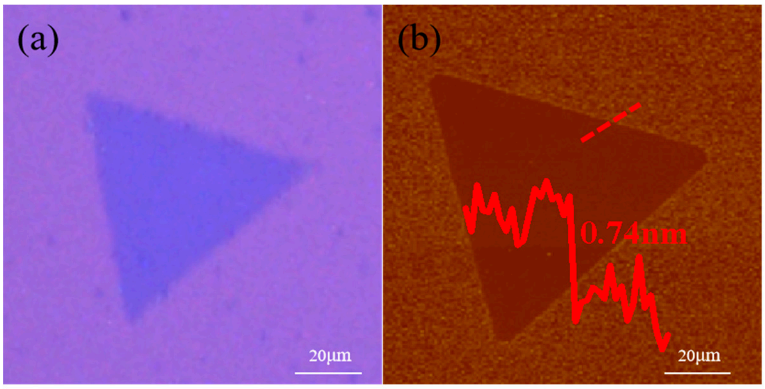

Figure S1. Topography of monolayer MoS_2. (a) light image. (b) AFM and step test.

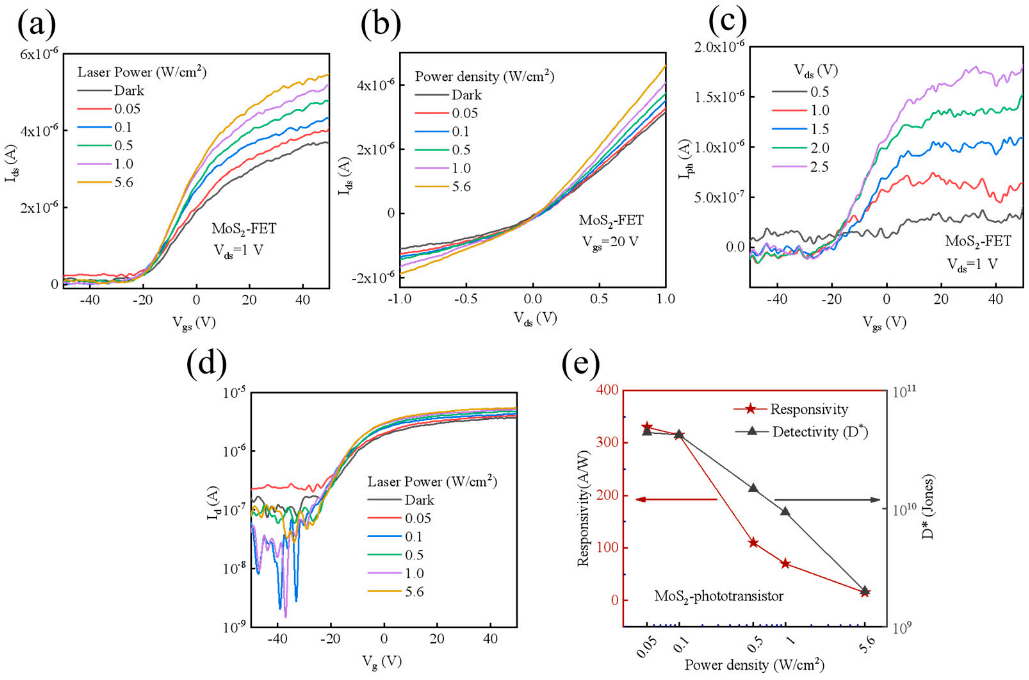

Figure S2. Device performance of bare MoS_2 phototransistor irradiating by a 405 nm laser with different power density. (a) Transfer characteristics with V_{ds}=1 V. (b) Output characteristics with V_{gs}=20 V. (c) The intrinsic photocurrent under light irradiation. (d) Photo-switching characteristics. (e) The calculated responsivity and detectivity.
